# Next-generation fertilizers: the impact of bionanofertilizers on sustainable agriculture

**DOI:** 10.1186/s12934-024-02528-5

**Published:** 2024-09-20

**Authors:** Pankaj Kumar Arora, Shivam Tripathi, Rishabh Anand Omar, Prerna Chauhan, Vijay Kumar Sinhal, Amit Singh, Alok Srivastava, Sanjay Kumar Garg, Vijay Pal Singh

**Affiliations:** 1 Department of Plant Science, Faculty of Applied Sciences, MJP Rohilkhand University, Bareilly, India; 2 Department of Environmental Microbiology, Babasaheb Bhimrao Ambedkar University, Lucknow, 226025 India; 3 Department of Law, MJP Rohilkhand University, Bareilly, India

**Keywords:** Nanofertilizer, Biofertilizer, Nanotechnology, Nanoparticle, Nanoencapsulation

## Abstract

Bionanofertilizers are promising eco-friendly alternative to chemical fertilizers, leveraging nanotechnology and biotechnology to enhance nutrient uptake by plants and improve soil health. They consist of nanoscale materials and beneficial microorganisms, offering benefits such as enhanced seed germination, improved soil quality, increased nutrient use efficiency, and pesticide residue degradation, ultimately leading to improved crop productivity. Bionanofertilizers are designed for targeted delivery of nutrients, controlled release, and minimizing environmental pollutants, making them a sustainable option for agriculture. These fertilizers also have the potential to enhance plant growth, provide disease resistance, and contribute to sustainable farming practices. The development of bionanofertilizers addresses the adverse environmental impact of chemical fertilizers, offering a safer and productive means of fertilization for agricultural practices. This review provides substantial evidence supporting the potential of bionanofertilizers in revolutionizing agricultural practices, offering eco-friendly and sustainable solutions for crop management and soil health.

## Introduction

As the world’s population is projected to reach 11 billion by 2100, food production needs to increase by 60–70% to meet the growing demand [1–3]. Traditional approaches to addressing food production challenges have involved the use of pesticides, chemical fertilizers, and genetically modified seeds [4].

The excess use of chemical fertilizers is reported to result in the degradation of soil health, reduction in food quality, and environmental problems [5]. A significant portion of these chemical fertilizers remains unused and can contribute to issues like leaching, mineralization, and bioconservation. The accumulation of chemical fertilizers has broader environmental consequences, affecting ecosystems such as soil microflora, marine environments, and parasites [6].

The challenges associated with traditional fertilizers, including issues with soil fertility, environmental impact, and inefficient nutrient utilization, have prompted a call for advancements in farming and agriculture [7–8]. One proposed solution is the implementation of nanotechnology, to achieve sustainable agriculture and enhance crop production [8–9].

Nanotechnology has a great potential for revolutionizing agriculture. By working at the ultra-small scale of nanoparticles (less than 100 nanometers in size), scientists are developing tools that can improve crop yields, fight disease, and promote sustainability [9–12]. Nanotechnology allows for targeted delivery of fertilizers, pesticides, and other agricultural products. By encapsulating them in nanoparticles, they can be delivered directly to the plant or target pest, reducing waste and environmental impact [13]. Nanofertilizers can deliver nutrients directly to plant roots, improving nutrient uptake and reducing waste. This can lead to healthier plants and improved crop yields [14]. Nanotech-based fertilizers can be designed to release nutrients slowly over time, ensuring optimal uptake by plants and reducing the need for frequent application. This can lead to increased crop yields and improved plant health. Nanotechnology can contribute to sustainable agriculture by reducing water and pesticide use [15–16]. Overall, nanotechnology has the potential to significantly improve agricultural practices, leading to increased food production, improved food safety, and a more sustainable food system.

Nanofertilizers and bionanofertilizers are two important nano-based products for sustainable agriculture that provide more efficient and targeted nutrient delivery systems to increase agriculture production [16–18]. The main objective of this review is to comprehensively assess the current state of knowledge and research on the application of nano-fertilizers and bionanofertilizers in agricultural systems. It aims to determine how these advanced fertilizers can support sustainable agriculture by boosting crop yields, enhancing soil quality, and reducing environmental harm. Additionally, the review intends to critically evaluate the effectiveness of bionanofertilizers in improving agricultural results in comparison to traditional fertilizers. This involves exploring their advantages, drawbacks, and future development possibilities. In this brief review, we discuss the synthesis, application, benefits, and limitations of bionanofertilizers, along with an overview of nanofertilizers.

## Nanofertilizers

Nanofertilizers are the kind on nanomaterials which act as plant nutrient (micro or macro nutrient) itself or carrier of the plant nutrients. Nutrients-encapsulated within nanomaterials are also known as nonofertilizers [19]. Nanofertilizers are nanomaterial-based formulations designed to deliver nutrients to plants in a more controlled and efficient manner. Nanofertilizers can be categorized into various types, including nano-sized particles of conventional fertilizers, nanostructured materials, and encapsulated nutrients that release slowly over time.

## Synthesis of nanofertilizers

The synthesis of nanofertilizers can be achieved through various methods, broadly classified into top-down and bottom-up approaches.

### Top-down synthesis

In this method of synthesis, bulk amount of material braked down into nano sized particles using various physical and chemical methods. Top-down synthesis includes, mechanical treatment, chemical treatment, thermal/laser extirpation, and sputtering [20]. In mechanical treatment large amount of material is break down into nano sized particle using ball-milling. Approximately 100 to 300 nm sized particles can be achieved by ball milling. In chemical treatment the materials are treated by various oxidizing or reducing agents such as NaBr, which changes the structure of the materials and leads to the formation of nano sized materials, which are further used as fertilizers. Thermal treatment synthesizes the material by carbon vapor deposition and treatment of the material till their reduction temperature for several hours in a particular gaseous environment [21]. In sputtering, thin films of nanoparticles are deposited using vacuum. A direct current magnetron sputtering process was optimized for producing uniformly sized silver nanoparticles [22].

### Bottom-up synthesis

**Fig. 1:**
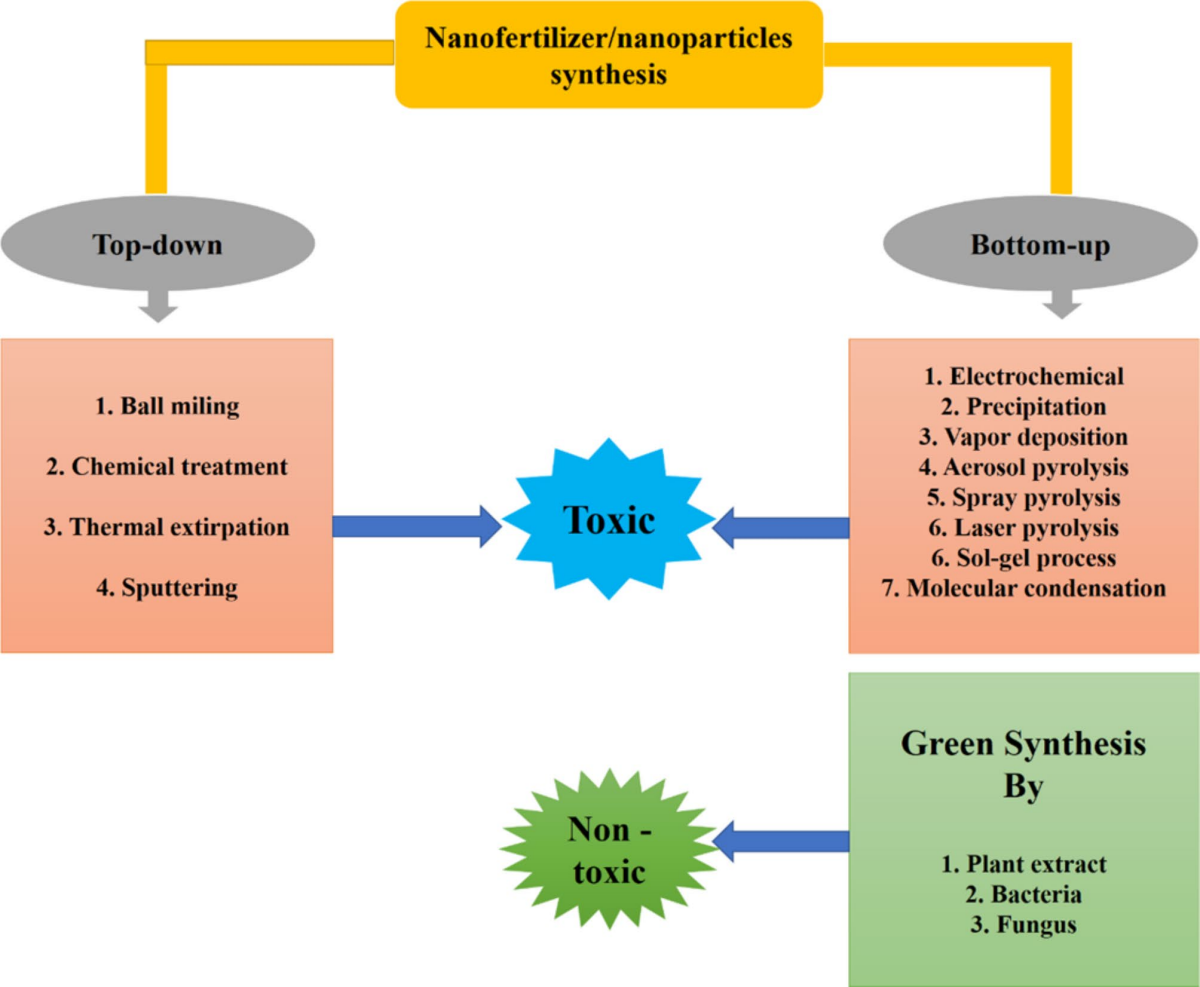
Synthesis methods of nanoparticles

In bottom-up synthesis nanofertilizers are synthesized from small monomers, atoms, and molecules. This approaches utilized physical and chemical forces at the nanoscale to assemble these building blocks into the desired nanoparticle structure [23]. The bottom-up synthesis performs using various chemical process such as precipitation, vapor deposition, sol-gel process, molecular condensation, laser pyrolysis, aerosol pyrolysis, and spray pyrolysis to synthesize nanoparticles [24] (Fig. 1).

### Biological synthesis

Biological synthesis of nanofertilizers involves the use of biological entities such as plants, microorganisms, and enzymes to produce nanoparticles [25, 26]. This method is also known as green synthesis. This method is often preferred over chemical synthesis due to its eco-friendliness, cost-effectiveness, and the ability to produce biocompatible materials.

## Types of nanofertilizers

Nano fertilizers can be classified into five major classes based on plant requirements and carrier support. These classes typically include macronutrient-based nanofertilizers, micronutrient-based nanofertlizers, Organic nanofertilizers, hybrid nanofertilizers aand carbon-based nanofertilizers. In this section, various types of nanofertilizers are discussed:

### Macronutrient-based nanofertilizers

Plants require a range of macronutrients to grow and flourish, including calcium (Ca), phosphorus (P), hydrogen (H), oxygen (O), magnesium (Mg), nitrogen (N), and sulfur (S). Insufficient amounts of these essential nutrients can hinder plant development and increase susceptibility to diseases and pests. Ensuring plants receive a proper balance of macronutrients is crucial for their health and vitality. The primary macronutrients, nitrogen, phosphorus, and potassium, are known as fertilizer elements and are indicated by the “NPK” labeling on fertilizer products [27]. As the demand for food rises towards 2050, the need for macronutrients will also grow. Nanofertilizers, which possess a high volume-to-surface ratio due to their nanomaterial composition, can enhance the efficiency of macronutrient uptake with smaller quantities compared to traditional fertilizers [28]. In this subsection, macronutrient-based nanofertilizers are discussed:

### Nitrogen-based nanofertilizers

Nitrogen is a crucial nutrient for plants due to its essential role in synthesizing amino acids, nucleic acids, and chlorophyll [29]. It is also a key element in enzymes that participate in energy metabolism, photosynthesis, and respiration. A deficiency in nitrogen leads to stunted growth, yellowing leaves, and reduced yield in plants [29]. Therefore, providing an adequate nitrogen supply is vital for healthy plant growth and productivity.

Nitrogen-based nanofertilizers incorporate nanoparticles (NPs) like metal oxides, graphene, and carbon nanotubes combined with nitrogen molecules. These fertilizers slowly release nitrogen into the soil, minimizing nitrogen runoff into aquatic systems and reducing the risk of environmental harm [15]. Research has shown that nitrogen-based nanofertilizers can enhance productivity more effectively than traditional mineral urea while mitigating its drawbacks. By increasing chlorophyll content in plant leaves, these nanofertilizers promote rapid growth in both shoots and roots [29]. With particles sized 20–50 nm, nano nitrogen particles have a higher surface area and more particles per unit area compared to conventional urea, and nano-urea may contain 4% nitrogen in its liquid form [29]. Researchers have also developed urea-coated zeolite chips and urea-modified hydroxyapatite nanoparticles, which provide a controlled, long-term release of nitrogen tailored to plant needs [30].

### Phosphorus-based nanofertilizers

Phosphorus is a vital mineral for plant development and growth, aiding in nutrient uptake and being essential for photosynthesis. Phosphorus nanofertilizers are more economical, efficient, and environmentally friendly compared to conventional fertilizers. Slow-release phosphorus nanofertilizers ensure a continuous supply of phosphorus throughout the crop’s life cycle. These nanofertilizers can quickly penetrate the plant cuticle through the cuticular pathway and travel long distances within the plant’s vascular system via the stomatal pathway. Research indicates that using phosphorus nanofertilizers can increase both grain and straw yield in Wheat [31]. This improvement is likely due to enhanced growth hormone levels, improved metabolic processes, and increased photosynthetic activity. The use of phosphorus nanofertilizers boosts photosynthesis and plant metabolism, leading to more panicles and grain development, ultimately resulting in higher wheat yields and better growth metrics [31]. In a study by Liu and Lal [32], apatite nanoparticles were applied as phosphorus nanofertilizer, and the seed yield and growth rate of *Glycine max* were improved by 20% and 30%, respectively, compared to conventional phosphorus fertilizers (Ca(H_2_PO_4_)_2_).

### Potassium-based nanofertilizers

Potassium (K) is one of the three primary macronutrients commonly used in agriculture, and its deficiency can negatively impact essential plant growth processes and crop yields. Potassium-based nanofertilizers exhibit enhanced nutrient uptake, reduced leaching susceptibility, and contribute to improved soil structure. A study on alfalfa plant (*Medicago sativa*) showed that potassium sulfate (K_2_SO_4_) nanoparticles enhanced growth, mineral content, and stress response mechanisms [33]. Similarly, daffodil plants (*Narcissus tazatta*) fertilized with potassium nanofertilizers exhibited significant differences in anthocyanin content in petals, biomass production, and water absorption [34].

### Calcium-based nanofertilizers

Calcium is vital for numerous functions, including stabilizing cell walls, retaining and transporting minerals in soil, neutralizing harmful compounds, and aiding seed development. It also plays a crucial role in fruit quality. As an intracellular messenger, calcium regulates stress signals, hormone responses, and various developmental processes. Calcium nanofertilizers have been shown to enhance crop yields, improve the quality of fruits and vegetables, and bolster plant disease resistance [15]. Nano-calcium carbonate has been shown to boost wheat photosynthesis by stimulating antioxidant enzyme activity, increasing the levels of photosynthetic pigments, enhancing Rubisco activity, improving stomatal conductance, and activating the PSII reaction center [35]. Furthermore, research indicates that the use of calcium nanofertilizers has led to a significant biomass increase of around 15% in peanuts (*Arachis hypogaea*) [36].

### Magnesium-based nanofertilizers

Magnesium is crucial for photosynthesis as it forms the core of the chlorophyll molecule, making it essential for plant growth. It also has the ability to activate enzymes [37]. Despite its importance, magnesium has been undervalued as a nutrient in recent years, earning it the moniker “the forgotten element”. Nano-Magnesium and phosphorus influenced atropine, hyoscyamine, and scopolamine levels in Datura seeds and leaves, as the PMT gene, which encodes the putrescine N-methyl transferase enzyme involved in atropine synthesis, is reduced in expression [38]. Foliar applications of nano-magnesium and chitosan to sesame plants subjected to water scarcity have the potential to influence the plants’ physiological functions and overall yield. These effects manifest in alterations to proline content, total sugar levels, the activities of peroxidase, catalase, and ascorbate peroxidase, photosynthetic pigment composition, seed yield, and oil content across different sesame cultivars [39].

### Sulfur-based nanofertilizers

Sulfur plays a crucial role in chlorophyll formation, enhancing nitrogen efficiency, and bolstering plant defenses [40]. Nanostructured sulfur-based growth enhancers have demonstrated an impact on the germination process of wheat seeds when cultivated in greenhouse and nursery environments. The application of nano-sulfur to sulfur-deficient soils enhances the efficiency of fertilizer utilization and accelerates the transfer of nutrients from plant vegetative parts to grain formation [41]. Cao et al. [42] demonstrated that sulfur nanoparticles measuring 30 nm and 100 nm were more effective in managing the *Fusarium* wilt than the use of bulk sulfur granules and sulfate. Studies have indicated that nano-sulfur serves as a plant growth enhancer, boosting stress resistance in plants and improving their nutritional quality [43]. Additionally, nano-sulfur can lower the uptake of toxic metals, thereby reducing food chain contamination and promoting food safety and security [44, 45].

### Micronutrient-based nanofertilizers

Micronutrients are trace elements which required in little amount (approximately ≤ 100 ppm), however they are essential for several metabolic process during plant growth. These micronutrients include Boron (B), Iron (Fe), Copper (Cu), Manganese (Mn), Zinc (Zn), Molybdenum (Mo), and Chlorine (Cl). In this subsection, we described micronutrient based nanofertilizers:

### Iron-based nanofertilizer

Iron is an essential micronutrient for plants, integral to key physiological processes such as chlorophyll production, respiration, and enzyme activity. Although iron is abundant in the Earth’s crust, it often occurs in forms that plants cannot easily absorb, resulting in deficiencies that can significantly reduce crop yields. Ghafariyan et al. [46] reported that an Iron nanofertilizer boosted chlorophyll content in *Glycine max* leaves by about 10%. Similarly, Delfani et al. [47] observed a 7% increase in seed weight in the *Vigna unguiculata* when using an iron-nanofertilizer. Application of Fe₂O₃ nanoparticles at a low dose (50 mg/L) to *Oryza sativa* (rice) enhanced crop growth. However, a higher concentration (500 mg/L) negatively affected growth, reducing root length, surface area, diameter, and volume [48].

### Copper-based nanofertilizer

Copper is a vital micronutrient for plants, essential for numerous physiological processes such as photosynthesis, respiration, and the production of enzymes and proteins. Copper nanoparticles increased the lignin content in the roots and reduced root growth in soybean plants [49]. Research has demonstrated that they are particularly effective in preventing various common bacterial and fungal diseases [50–51].

### Boron-based nanofertilizer

Plants require small amounts of boron as a fertilizer element, but it is essential for developing cell walls and for transporting photosynthesis products from leaves to active growth regions. While fertilizers can help alleviate boron deficiency, frequent application can negatively impact soil fertility and the environment. Boron is also crucial for bark development, the transport of hormones that affect stem and root growth, pollen germination, flowering, and increasing carbohydrate delivery to active growth areas during the reproductive stage. Meier et al. [52] documented increases in both root and aerial biomass in lettuce and zucchini plants uning Boron nanofertilizrs. In contrast, Davarpanah et al. [53] discovered that applying a boron (B) nanofertilizer enhanced the yield of pomegranate trees and, when used with a zinc nanofertilizer, improved fruit quality. Similarly, Ibrahim et al. [54] found significant improvements in plant height, pod number, and overall yield in mung bean (*Vigna radiata* L.) plants when boron nanoparticles were applied foliarly.

### Mangenese-based nanofertilizer

Manganese nanoparticles have been demonstrated to be a more effective source of manganese micronutrients than the commercially available MnSO₄ salt [55]. MnNPs not only support plant growth but also enhance photosynthesis in mung beans (*Vigna radiata*). In contrast, MnSO₄ salt inhibited plant development at a concentration of 1 mg/L, while MnNPs maintained a positive impact. Specifically, at a concentration of 0.05 ppm, Mn nanofertilizer increased root, shoot, rootlet, and biomass growth in *Vigna radiata* by 52%, 38%, 71%, and 38%, respectively [56].

### Nickel-based nanofertilizer

Nickel is a trace element essential for various plant functions, such as enzyme activation, nitrogen metabolism, and chlorophyll synthesis. In a study with 10-day-old wheat seedlings, nickel nanoparticles (NPs) at low concentrations of 0.01 and 0.1 mg/L did not markedly affect growth or development. However, a small increase in chlorophyll a and chlorophyll b content was noted with the 0.01 mg/L application [57]. Despite its trace element status, nickel’s absorption is crucial for numerous enzyme functions, cellular redox regulation, and several development-related processes, including growth and physiological and biochemical responses.

### Zinc-based nanofertilizers

Zinc nanoparticles (ZnNPs) have been demonstrated to significantly enhance various plant growth parameters, including root and shoot length, biomass, and overall yield. In plants such as *Vigna radiata*,* Cicer arietinum*,* Cucumis sativus*,* Raphanus sativus*,* Brassica napus*, and Cluster bean, ZnO nanofertilizers have notably improved biomass, shoot and root length, chlorophyll content, protein levels, and phosphatase enzyme activity [58, 59]. ZnNPs also boost plant tolerance to a range of stresses, such as drought, salinity, and heavy metal toxicity, by helping plants maintain a more balanced physiological and biochemical state under stress. ZnNPs can increase chlorophyll content and photosynthetic activity, leading to improved growth and higher crop yields.

### Organic nanofertilizers

Organic nanofertilizers consist of naturally occurring substances, derived from plant, animal, or mineral sources, that are engineered to nanoscale dimensions (1–100 nm). They are derived from organic matter like compost, manure, or plant residues or polymers such as chitosan, alginate, carrageenan, pectin etc [60].

One promising example of organic nanofertilizers is the use of chitosan-based nanofertlizers [61]. Chitosan, a natural polymer derived from chitin, forms a biodegradable and biocompatible matrix that can encapsulate various nutrients. To date, several types of chitosan-based nanpfertilizers have been developed including nanochitosan, nanochitosan nano-NPK fertilizers and Cu-chitosan [61]. Another example is NanoMax-NPK that contains organic micronutrients/trace elements, vitamins, and probiotics, along with multiple organic acids (protein-lacto-gluconates) including include chelated nitrogen, phosphorus, potassium, oxygen, amino acids, and organic carbon [60]. Ferbanat and nanonat are examples of liquid organic fertilizers that enhanced growth of the yield of cucumbers [62].

Georgieva et al. [63] assessed the effects of two organic nanofertilizers, Lithovit and Nagro, on in vitro germination, pollen tube elongation, and pollen grain viability in *Pisum sativum* L. cv. Pleven 4. The application of these nanofertilizers significantly improved results compared to the untreated control, with increases of 44.2% and 47.23% in pollen germination and pollen tube elongation, respectively.

Organic nanofertilizers represent a significant advancement in sustainable agriculture. They offer efficient nutrient delivery, improved soil health, and enhanced plant growth, contributing to higher yields and better environmental outcomes.

#### Hybrid nanofertilizers

Hybrid nanofertilizers are innovative agricultural products combining the strengths of different nanomaterials to deliver essential nutrients to plants in a controlled and efficient manner [64]. These fertilizers offer a promising solution to address the challenges of traditional fertilizers, such as nutrient loss, environmental pollution, and low crop yields.

Hybrid nanofertilizers typically comprise a nanomaterial carrier, essential nutrients, and often additional components such as growth regulators. The nanomaterial carrier, often composed of silica or polymer nanoparticles, encapsulates the nutrients, protecting them from degradation and facilitating controlled release [64]. This controlled release mechanism ensures a sustained supply of nutrients to the plant, optimizing uptake and minimizing losses through leaching.

Tarafder et al. [65] introduced a novel, hybrid nanofertilizer designed for the gradual and sustained release of nutrients into soil and water environments. This innovative fertilizer is constructed from urea-modified hydroxyapatite, that supplies essential nutrients including nitrogen, calcium, and phosphorus. To further enhance the fertilizer’s effectiveness, nanoparticles of copper, iron, and zinc were incorporated into the urea-modified hydroxyapatite matrix, thereby improving nutrient delivery and overall efficiency. The hybrid fertilizer significantly boosted the absorption of copper, iron, and zinc nutrients in okra plants (*Abelmoschus esculentus*) due to the slow release of these elements from this hybrid fertilizer.

Haruna et al. [66] produced a nanohybrid urea-hydroxyapatite fertilizer loaded with carbon nanotube. The inclusion of carbon nanotubes likely helps in maintaining the structural integrity of the fertilizer, which in turn ensures a more controlled and prolonged release of nutrients.

### Carbon-based nanofertilizers

Carbon-based NFs have a widespread use in the development of plant and enhances the growth of plant [67]. Carbon NFs Effectively passing through the seed coat and moving from the root to the shoot and leaf, and can move throughout plants. When nanocarbons suspended or distributed to water and growth medium, then the plants can easily absorb them while taking in other essential minerals. These nanocarbons are mainly utilized by the roots of treated plants to enhance their ability to transport water. Carbon nanotubes have emerged as potential agricultural enhancers, demonstrating their ability to boost growth and yield in various plants [67]. Studies have indicated that these materials can be effectively applied as fertilizers for a wide range of crops, including vegetables like tomato, cabbage, carrot, rape, onion, and cucumber, as well as agricultural staples such as soybeans, ryegrass, and corn [68, 69].

A key mechanism behind this enhancement is the ability of carbon nanotubes to facilitate water absorption by plants. These structures can penetrate germinating seeds, stimulating growth [70]. Moreover, research on multi-walled carbon nanotubes (MWCNTs) has revealed their efficacy in improving seed germination for a diverse array of crops, including tomatoes, corn, and garlic [69. 72]. Khodakovskaya et al. [72] have shown that MWCNTs can penetrate tomato seeds, leading to increased water uptake and subsequently higher germination rates. Their experiments demonstrated a remarkable 90% increase in seed germination compared to untreated seeds after a 20-day treatment period.

### Mechanism of plants uptake of the nanofertilizers

The ability of nanofertilizers to transport nutrients to target areas in biological systems is quite promising. Plants can absorb and move nanoparticles, and several variables, including particle size, surface charge, concentration, exposure duration, and plant type, influence this process. Some entry points, including stomata, root hairs, and surface fractures on leaves, allow nanoparticles to enter the plant system. After entering the plant, nanoparticles can propagate by bulk flow, phloem loading, and diffusion throughout the plant system. Numerous variables, including the size, shape, and surface characteristics of the nanoparticles, as well as the pH and presence of other ions or chemicals in the solution, might affect how well the nanoparticles move.

### Uptake of nanofertilizers through plant leaves (Foliar mode)

Nanofertilizers are sprayed on the leaves of the plants or crops. Nanofertilizerts deposited on the surface of the leaves and after deposition they get absorbed through the stomata present on the leaf or by the cuticle [73]. The primary constituents of the waxy cuticle found on leaf epidermis are wax, cutin, and pectin. The waxy stratum corneum has two distinct channels on its surface: one is lipophilic and the other is hydrophilic. Both the lipophilic and hydrophilic channels have a range of sizes between 0.6 and 4.8 nm. Hydrophilic nanoparticles with a diameter smaller than 4.8 nm can easily diffuse through the hydrophilic channels. On the other hand, lipophilic nanoparticles are absorbed by the leaf through infiltration and diffusion due to the presence of lipophilic channels in the cuticle surface. For plants to regulate the exchange of gases and water, the stomata on their leaf surfaces are essential. Typically, stomata range in size from 10 to 100 μm. Different plant species differ in the size and density of their stomata. The precise size exclusion limit of the stomatal aperture for nanoparticle diffusion is yet unknown because of the distinct geometric structure and physiological role of stomata [73].

### Uptake of nanoparticles through plant roots

Adsorption takes place on the root surface to initiate the first interaction between nanoparticles and plant roots. Since organic acids and mucus can be released by the root hairs, the root surface is negatively charged. This means that positive-charged nanoparticles are more likely to aggregate in the root and be readily absorbed on the root surface. Nanoparticles may be able to reach the root column through the development of lateral roots, which may open up a new adsorption surface for them. The plant root epidermis resembles the plant leaf surface in terms of composition and function. However, the surface of the primary and secondary roots’ root hair and plant root tip epidermis are not completely grown. Upon exposure, the root epidermis is directly affected by the nanoparticles, which then penetrate it. There are several methods that plant cells can absorb nanoparticles when they get into their tissue, including the ion route, endocytosis, interaction with cell membrane. After absorption, nanoparticles can move through symplastic and apoplastic pathways [74].

### Interaction of nanofertilizers with plant cells

Nanoparticles are atomic aggregates with at least one dimension between 1 and 100 nm. Their physicochemical properties differ from larger materials due to their high surface area-to-volume ratio, resulting in unique biological, chemical, and physical properties. Various imaging techniques, including Transmission Electron Microscopy (TEM) and confocal microscopy, have been used to demonstrate how nanoparticles interact with plant cells and tissues. The unique characteristics of nanoparticles, such as small size, high surface-to-volume ratio, and electron exchange ability, contribute to these interactions [75].

Plants can absorb nanoparticles through different routes, including stomatal, lipophilic, cuticular, and hydrophilic pathways when applied to leaves. The size of nanoparticles influences the absorption route, with smaller nanoparticles (0.6–4.8 nm) absorbed by hydrophilic, cuticular, and lipophilic pathways, while larger nanoparticles (over 20 nm) can pass through stomata [76].

**Fig. 2:**
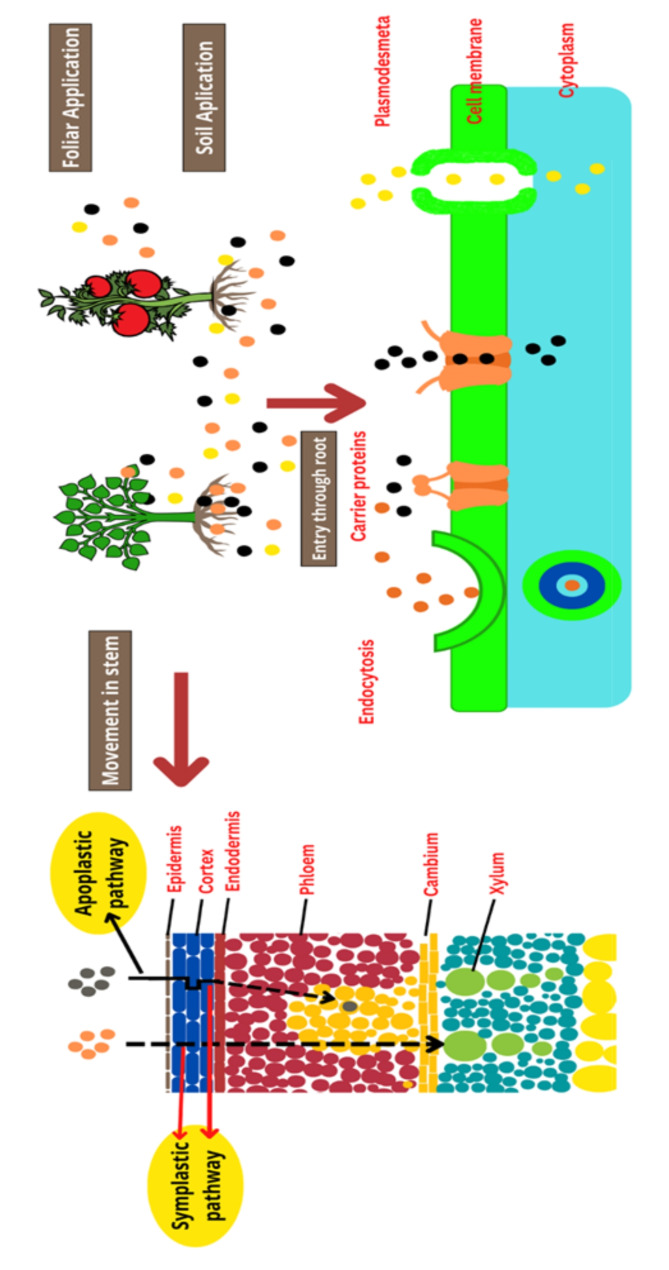
Uptake and translocation of nanofertilizers inside the plant cells

The phloem system is identified as the primary pathway for nanoparticle transmission from leaf surfaces to roots, as macromolecules and nutrients move through the phloem [77]. Various transport mechanisms, including aquaporins, endocytosis, ion channels, and membrane transporters, are involved in nanoparticle transport [78–79]. It is mentioned that polymer-coated nanofertilizers act as smart nanofertilizers that release nutrients in a controlled manner [80]. Formulating nanofertilizers with polymers such as alginate, albumin, chitosan, and polyacrylates, polycaprolactones, and polylactide is recommended [17]. Nanofertilizers, such as chitosan-coated nanofertilizers and nanoclay-based fertilizers, have shown increased effectiveness in releasing nutrients compared to conventional fertilizers. Natural zeolites and urea-hydroxyapatite hybrid nanofertilizers are also mentioned as effective in binding and gradually releasing nutrients in soil [81]. A range of channels, including apoplastic, symplastic, and transmembrane, are used to absorb nano fertilizers by roots. The size of particles that can be absorbed by plants ranges from 7 to 200 nm. The positive surface charge of nanoparticles contributes to their absorption onto the negatively charged root surfaces. The distribution and accumulation of nanofertilizers in crop plants are influenced by factors such as particle characteristics, surrounding conditions, plant type, and rhizosphere composition. The application method plays a crucial role in determining the effectiveness of nanofertilizers for plant growth and development [81]. Figure 2 explains mode of uptake of nanofertilizers through various mechanism.

### Significance of nanofertilizers

The conventional fertilizers suffer from significant nutrient loss due to leaching and volatilization [15]. Nano-fertilizers address this by encapsulating nutrients within a nanoscale shell, enabling controlled release and direct delivery to plant roots [82]. This improved nutrient use efficiency can lead to a reduction in the overall amount of fertilizer required. Studies have shown that controlled release nanofertilizers can significantly enhance crop productivity [83]. Research demonstrated improved crop yield, nutrient use efficiency, and overall plant health in various crops like rice, wheat, corn, and soybeans [84]. By ensuring a steady supply of essential nutrients, nano-fertilizers promote plant growth and development, leading to larger and potentially higher quality crops.

Plants treated with nano-fertilizers often exhibit a stronger defense system against pathogens and pests [85]. This can be attributed to improved nutrient uptake, leading to the production of natural defense compounds and activation of stress response pathways within the plant. Certain types of nanofertilizers, like those containing copper or zinc oxide, possess inherent antimicrobial properties [86]. These nanoparticles can directly target and inactivate harmful bacteria, fungi, and viruses, reducing disease incidence in crops.

As mentioned before, nanofertilizers enhance nutrient uptake by plants. This improved nutritional status makes plants more resilient to abiotic stresses such as drought, salinity, and extreme temperatures.

Studies suggest that nano-fertilizers can stimulate the production of antioxidants within plants [87]. These antioxidants help scavenge harmful free radicals generated during stress conditions, protecting plant cells and tissues from damageNano-fertilizers might influence the production of plant hormones like auxins and cytokinins, which play a vital role in stress tolerance [88]. By promoting optimal hormone balance, these nanoparticles can help plants better adapt to unfavorable environmental conditions [89].

Abiotic stresses like drought or high temperatures can hinder traditional fertilizer uptake. Nano-fertilizers with their controlled release mechanism can ensure a steady supply of nutrients even under stressful conditions, supporting plant resilience. Table 1 summarizes role of various nano fertilizers in plant growth under various stress and non-stress conditions.

**Table 1:** Role of various nano-fertilizers in plant growth under various stress and non-stress conditions

S. No	Nano fertilizer	Crop name	Impact	Reference
1	Mg	*Vigna unguiculata*	Enhancement in Mg content of stem, a higher chlorophyll content and a more stable plasma membrane	[47]
2	Cu	*Lactuca sativa*	Increment in shoot and root length	[90]
3	Ca	*Arachis hypogaea*	enhanced nutrient content in shoots and roots	[36]
4	Au	*Pennisetum glaucum*	Enhanced germination of seeds and growth of seedlings	[24]
5	CeO_2_	*Cucumis sativus*	Enhancement in scratch and globuline content	[91]
6	Fe/SiO_2_	*Hordeum vulgare and Zea mays*	Mean germination time improved	[92]
7	CNTs	*Phoenix dactylifera*	Increment in shoot length and number of leaf	[93]
8	CuO	*Zea mays*	Approximately 51% increased plant growth	[94]
9	FeO	*Glycine max*	Chlorophyll content increased	[47]
10	Mn	*Vigna radiata*	In addition to increasing chlorophyll content and shoot length, photosynthesis rate also increased	[56]
11	Zn	*Lolium*	Elongation of root	[58]
12	Mo	*Cicer arietinum*	Increment in number of modules and plant mass	[95]
13	TiO_2_	*Spinacia oleracea*	Dry weight of plant increased.	[96]
14	P	*Glycine max*	There was a 32.6% increase in growth rate and a 20.4% increase in seed yield	[19]
15	TiO_2_	*Glycine max*	Enhanced germination characteristics, reduced H_2_O_2_ and MDA content, and increased DPPH free radical scavenging in soybean and increased salt tolerance	[97]
16	Mesoporous silica nanoparticle	*Arabidopsis thaliana*	Induced drought tolerance	[98]
17	Fe	*Brassica napus*	Regulated succinate dehydrogenase activity under drought stress	[99]
18	Silica Nanoparticles	*Triticum aestivum*	Upregulated stress genes to remove effects of drought	[100]
19	Chitosan	*Catharanthus roseus*	Genes like DAT, STR, PRX1, and GS had increased expression, and there was also an increased alkaloid content.	[101]
20	Cu	*Zea mays*	Increased plant growth and grain yields	[102]
21	FeO	*Triticum aestivum*	Increased 37% growth in stress of cadmium and salinity	[103]
22	Magnetite	*Triticum aestivum*	Increased chlorophyll content, growth, glutathione, and soluble proteins to counteract salt stress	[104]
23	ZnO	*Trigonellafoenum-graecum*	Under high salinity stress, the activities of various stress enzymes significantly increased	[105]
24.	Fe	*Triticum aestivum*	Under cadmium and drought stress, iron nano particles improved the yield, photosynthesis, Fe concentrations and diminished the Cd concentrations in tissues	[106]
25.	Ag	*Glycine max*	Under flooding stress, root length/weight and hypocotyl length/weight of soybean were enhanced by silver NPs mixed with nicotinic acid and KNO_3_.	[107]
26.	Se	*Pisum sativum*	Under salinity stress, the combined treatment of nano-selenium, ascorbic acid, 5-aminolevulinic acid on pea seedlings removed damaging effects of salinity stress and improved seedling performance	[108]
27.	CeO_2_	*Gossypium hirsutum*	Improved root system under salinity stress	[109]
28.	SiO_2_	*Musa acuminata*	Under drought stress, removed oxidative stress. Decreased cell damage and increased chlorophyll content	[110]
29.	S-nitrosoglutathione encapsulated chitosan nanoparticles	*Saccharum officinarum*	Increased growth of sugarcane under drought conditions	[111]
30	Chitosan	*Triticum aestivum*	Mitigated Drought-related adverse effects. Induced activities of superoxide dismutase and catalase, Significantly increased chlorophyll content, photosynthesis rate, leaf area, relative water content, yields, and biomass.	[112]
31.	Fe	*Fragaria ananass*	Removed harmful effects of the drought. Improved quality and quantity of the strawberry plantlets	[113]
32	ZnO	*Solanum lycopersicum*	Enhanced glutathione peroxidase as well as superoxide dismutase under drought-related conditions	[114]
33.	Al_2_O_3_	*Glycine max*	Affected mitochondrial proteins under flooding conditions. Regulated tricarboxylic acid cycle and membrane permeability	[115]
34	Ni	*Bean*	Ni content increased. Showed positive effects on physiological characteristics	[116]
35.	ZnO	*Potato*	Increased the tuber quality and Zn content but reduced the total number of potato tuber	[117]
36.	Mn (MnO_2_ and Mn_3_O_4_	*Radish*	Vitamin C and sugar content increased. However, uptake of elements like Cu. Fe. Mg, Zn. Na and K were suppressed	[118]
37.	ZnO	*Mealybug* *(Puto* *barberi)*	Showed 55% effectiveness on the pest population	[119]

### Bionanofertilizers

Biofertilizers are a type of fertilizer that contains living microorganisms that promote plant growth by increasing the availability of nutrients in the soil. These microorganisms include bacteria, fungi, and algae, all of which play a vital role in enhancing soil fertility and plant health [120]. Biofertilizers contribute to the soil by fixing atmospheric nitrogen, solubilizing phosphorus, and mobilizing other essential nutrients that are often unavailable to plants in their natural state. This ensures that plants have a steady supply of the nutrients they need to thrive [121–123].

Despite the benefits, traditional biofertilizers face challenges such as poor shelf life, sensitivity to environmental conditions (pH, radiation, temperature), on-field stability issues, and the need for large quantities for extensive agricultural areas [124]. To overcome these problems, bionanofertilizers have been synthesized by combining biofertilizers with nanoparticles [125, 126].

Bionanofertilizers represent innovative approaches to enhance nutrient delivery to plants and improve agricultural productivity [81, 125]. These technologies leverage nanotechnology to create more efficient and targeted nutrient delivery systems. Nanoencapsulation of biofertilizers is one of the techniques used in formation of bionanofertilizers [17]. Nanoencapsulation, involving the coating of biofertilizer components in nanoscale polymers, is highlighted as a technique to protect components, extend shelf life, and ensure controlled release of plant growth-promoting microorganisms (PGPR). Bionanofertilizers offer several advantages, including steady and slow nutrient release to plants, improved field performance, reduced application losses, cost-effectiveness, eco-sustainability, and renewability [17]. It is suggested to accelerate nutrient uptake (NPK), enhance enzyme activity, increase microbial populations beneficial for the soil, improve soil fertility, enhance crop product quality, and make crops more resistant to diseases. The potential benefits of bionanofertilizers include enhanced agricultural productivity, sustainability, and food security [126].

### Biosynthesis of bionanofertilizer

**Fig. 3:**
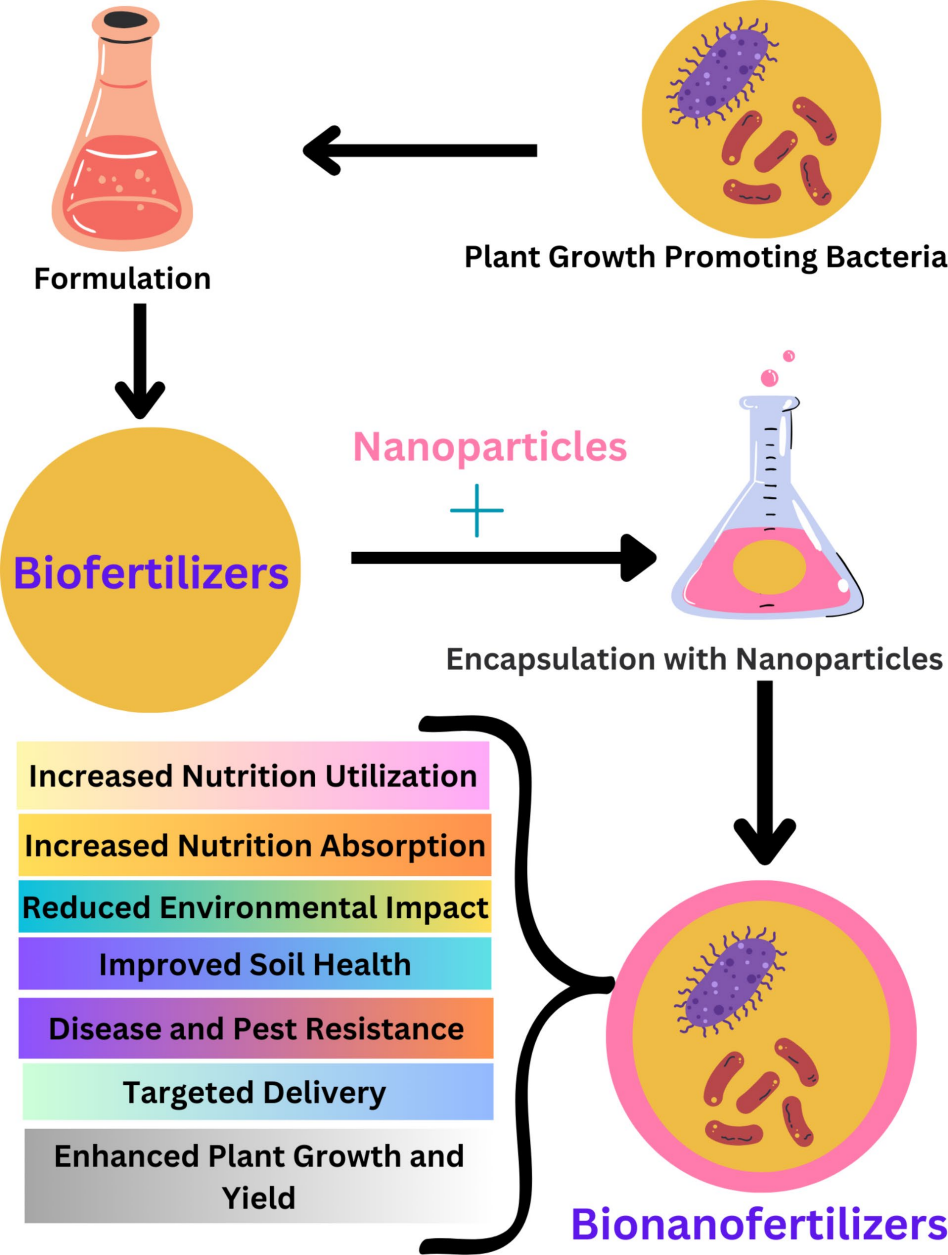
Synthesis and Applications of bionanofertilizers

The synthesis of bionanofertilizers involves three key steps: (1) cultivating the biofertilizer culture; (2) encapsulating it with nanoparticles; and (3) evaluating its efficacy, quality, purity, and shelf life [74, 127] (Fig. 3). An alternative method for producing bionanofertilizer includes the formation of microcapsules. PGPR suspensions are mixed in a 2:1 ratio with sodium alginate, starch, and bentonite to produce PGPR mixtures crosslinked with calcium chloride solutions, followed by sterile distilled water washing of the formed microcapsules [128]. Additionally, a bionanofertilizer can be created with the reaction of salicylic acid with nanoparticles. Sodium alginate (2%), ZnONPs (1 g/mL), and salicylic acid (1.5 mM) are added to the biofertilizer in this method. A 3% calcium chloride solution is then applied to the mixture, resulting in beads of 1 mm. These beads are air-dried and incubated at 4 °C [127]. An effective bionanofertilizer can be produced by combining organic wastes, such as flowers, cow dung, and kitchen waste, with nanoparticles. Decomposition or pyrolysis of organic waste is performed after the waste has been washed to remove impurities. To produce bionanofertilizer, partially decomposed or pyrolyzed waste is combined with nanoparticles [129].

The development and implementation of bionanofertilizer formulations on a large scale face limitations due to a lack of complete understanding of the interactions between nanoparticles, biofertilizers, microflora, and plant systems.

Hamed et al. [130] developed novel bionanofertilizer capsules designed for the slow and controlled release of nutrients (NPK) and the plant growth-promoting bacterium, *Pseudomonas fluorescens*. These capsules were created by crosslinking chitosan and alginate with humic acid, followed by the incorporation of nano-NPK and PGPR. The application of these capsules demonstrated that they effectively delivered NPK in a controlled manner, potentially enhancing agricultural productivity and sustainability.

### Applications of bionanofertilizers

There are two methods for applying nanofertilizers to crop plants: independently as biofertilizers and nanofertilizers or jointly as a bionanofertilizer. When used independently, nanoparticles can directly impact plant growth by enhancing various processes such as photosynthesis, carbon sequestration, seed germination, enzyme activity, and nitrogen fixation. Using the microbe *Pseudomonas aeruginosa*, Shukla et al. [131] reported that nanoparticles enhanced the efficacy of biofertilizers significantly increasing crop yields and nutritional value. Celsia and Mala [132] investigated the role of neem cakes and PGPR in enhancing the growth and survival of *Vigna radiata* seeds using nanostructured NPK fertilizers. Rajak et al. [133] demonstrated that using a combination of bionanofertilizer with other nanofertilizers, such as copper nanoparticles (CuNPs), can effectively encourage plant growth and vitality. Farnia and Omidi [134] observed a notable improvement in grain output (about 1.5–2 times) in *Zea mays* crops after applying bionanofertilizer (nano-Zn + biofertilizer) for 7 days. Sabir et al. [135] conducted experiments to compare the effectiveness of nanofertilizer alone versus its combination with biofertilizer (*Ascophyllum nodosum*) on grapevine plants. The combined application showed a notably high contribution to improvements in vine growth, yield, berry quality attributes, and leaf nutrient levels, particularly in alkaline soil conditions. Mukhopadhyay and De [136] found that nanoclay-coated biofertilizer, containing *Trichoderma* and *Pseudomonas* sp., improved the water retention capacity and nutrient utilization efficiency of rainfed Rabi crops, leading to increased crop productivity. Rahman and Zhang [137] explored the biochemical processes promoted by bionanofertilizer application, suggesting that it improves the yield characteristics of crops like *Vigna*.

Moradi Pour et al. [138] nanoencapsulated *Bacillus velezensis* with sodium alginate and geletin for biocontrol of *Pistachio gummosis*. Jakien et al. [139] studied sugar beet plants and found that bionanofertilizer had significant potential for enhancing morphological and physiological characteristics, including leaf area, net photosynthetic productivity, root biomass, and sucrose content. This resulted in an increased yield of white sugar. Safaei et al. [140] investigated the effectiveness of bionanofertilizer (nanopharmax + humic acid) on black cumin (*Nigella sativa*). The study revealed that the bionanofertilizer increased the amount of nutritious elements in *N. sativa*. Mir et al. [141] investigated the impact of bionanofertilizers on the nutrients, carbohydrates, and pigment content of various plants, particularly forage sorghum. By using these fertilizers, agricultural crops obtained significant nutrient enhancements, chlorophyll increases, and carbohydrate gains. More recent applications of bionanofertilizers are summarized in Table 2.

Overall, these studies collectively demonstrate the potential benefits of bionanofertilizers in improving crop growth, yield, nutrient absorption, and various physiological characteristics across different plant species and environmental conditions.

**Table 2:** Applications of different kinds of bionanofertilizers

Plants	Bionanofertilizer	Effect on Plants	References
*Oryza sativa*	*Bacillus* sp. MR-1/2 with magnetite nanoparticles	Reduced oxidative stress and increased absorption of N in rice grown in conditions of water scarcity	[142]
*Cajanus cajan*	*Piriformospora Indica* with copper nanoparticles	Maximum vigour and healthy seedlings	[133]
*Brassica napus*	*Bacillus amyloliquefaciens* strain UCMB5113 with titania nanoparticles	Prevented infection and boosted *Bacillus amyloliquefaciens* UCMB5113’s adherence to roots.	[143]
*Zea mays*	Nano-potassium fertilizer, humic acid and compost manure	Notable improvement in maize grain yield and quality	[144]
*Triticum aestivum*	*Bacillus aryabhattai* RSO25 with iron nanoparticles	Iron accumulation in spikes.	[145]
*Pistacia vera*	Sodium alginate*-* encapsulated *Bacillus velezensis* + carbon nanotubes + silicon nanoparticles	As a biocontrol agent, nano formulations provided protection for PGPR against unfavourable environmental circumstances.	[138]
*Citrullus lanatus* *Vigna radiata*, *Brassica nigra*	*Chlorella* K01 and Iron nanoparticles	Boosted plant development and resistance against several fungal diseases.	[146]
*Zea mays*	*Bacillus cereus* LPR2 and silver nanoparticles	Induced plant growth and inhibited fungal pathogen.	[147]
*Zea mays*	*Pseudomonas +* Mycorrhiza *+* Iron nano-oxide	Increased growth normal and drought stress	[148]
*Vigna unguiculata*	*Pseudomonas monteilii +* chitosan nanoparticles	Increased shoot length, and fresh weight along with leaf number,	[149]
*Melissa officinalis*	*Pseudomonas fluorescens*,* P. putida +* Silicon	Enhanced primary and secondary metabolites	[150]
*Cicer arietinum*, *Triticum aestivum*	*Panebacillus polymyxa +* acetylated cylated homoserine lactone	Improved growth and pathogen defence	[151]
*Phaseolus vulgaris*	Titanium nanoparticles enriched Alginate – bentonite coated *Bacillus subtilis*	Control pathogen *Rhizoctonia solani*	[152]
*Trifolium repens*	*Pseudomonas* *fluorescens +* Zero valent iron nanoparticles	Increased Antimony phytoremediation	[153]
*Brassica juncea*	Biochar + compost + ZVI nanoparticles	Increased heavy metal remediation	[154]
*Triticum aestivum*	Zinc oxide nanoparticles with composted organic amendments	Increased cadmium accumulation	[155]
*Triticum aestivum*	Zinc oxide nanoparticles *+ Bacillus* spp.	Increased nitrogen content	[156]

### Advantages of biofertilizers

Bionanofertilizers offer a groundbreaking approach to boosting agricultural output while preserving the environment. By merging the benefits of biological agents and nanotechnology, these innovative fertilizers tackle key agricultural challenges. This section delves into the potential of bionanofertilizers to transform farming practices.

### Enhanced nutrient utilization

One of the key benefits of bionanofertilizers is their capacity to enhance nutrient utilization. Traditional fertilizers often encounter challenges with nutrient leaching and poor plant uptake. Bionanofertilizers tackle these issues by employing nanoparticles to deliver nutrients in a controlled and sustained manner. This controlled release system ensures a steady supply of nutrients to plants, minimizing waste and increasing overall efficiency [157]. Bionanofertilizers containing phosphorus-loaded nanoparticles can significantly improve phosphorus utilization. The nanoparticles gradually release phosphorus, making it readily available to plants. Additionally, some nanoparticles can interact with the soil to enhance phosphorus solubility, further increasing its availability [157]. Certain bionanofertilizers may contain phosphorus-mineralizing bacteria mineralizes organic phosphorus into plant-available forms.

### Increased nutrient absorption

Bionanofertilizers optimize nutrient uptake by increasing the availability of nutrients, improving their solubility, and facilitating their transport into plant cells. Furtheremore, nanobiofertilizers containing beneficial microorganisms can enhance nutrient uptake by promoting the formation of mycorrhizal networks, which facilitate the transport of nutrients from the soil to plant roots [158].

### Reduced environmental impact

Environmental sustainability is a critical concern in agriculture, and bionanofertilizers offer several advantages in this regard. Conventional fertilizers often lead to nutrient runoff, which can cause water pollution and eutrophication. Bionanofertilizers mitigate these issues by improving the efficiency of nutrient use and reducing the potential for nutrient leaching. Nanoparticles in bionanofertilizers can enhance nutrient retention in the soil, leading to reduced environmental impact and lower risk of water contamination. Additionally, the use of bionanofertilizers can contribute to the reduction of greenhouse gas emissions associated with traditional fertilizers [159].

### Improved soil health

Bionanofertilizers contribute to improved soil health by enhancing soil microbial activity and structure. Many bionanofertilizers contain beneficial microorganisms that promote soil fertility and health [158]. These microorganisms can improve soil aeration, increase organic matter content, and stimulate beneficial microbial communities. The interaction between nanoparticles and soil microorganisms can lead to enhanced soil microbial diversity and activity, which is essential for maintaining healthy and productive soils.

### Targeted delivery and customization

The nanoscale design of bionanofertilizers allows for targeted delivery of nutrients and customized formulations. Unlike traditional fertilizers, which may spread nutrients indiscriminately, bionanofertilizers can be engineered to deliver nutrients precisely to the plant roots or specific soil areas. This targeted approach enhances the efficiency of nutrient use and reduces wastage [15]. Furthermore, the ability to customize bionanofertilizer formulations based on specific crop requirements and soil conditions allows for more effective and tailored fertilization practices [15].

### Reduced dosage and cost savings

Bionanofertilizers often require smaller quantities compared to conventional fertilizers due to their enhanced efficiency. The improved nutrient release and absorption capabilities of bionanofertilizers mean that lower doses can achieve the same or better results as larger amounts of traditional fertilizers. This reduction in dosage not only lowers costs for farmers but also minimizes the environmental impact associated with excessive fertilizer application [15].

### Enhanced plant growth and yield

Bionanofertilizers have been shown to significantly enhance plant growth and yield. By improving nutrient availability and absorption, bionanofertilizers can lead to better growth parameters such as root development, shoot height, and leaf area. Research has demonstrated that the application of bionanofertilizers can result in increased crop yields and improved quality across a range of crops, including cereals, vegetables, and fruits [133]. This enhancement in plant growth contributes to higher productivity and better crop performance.

### Disease and pest resistance

In addition to their role in nutrient supply, some bionanofertilizers also offer benefits in terms of disease and pest resistance [152]. Certain bionanofertilizers have demonstrated antimicrobial properties that can help protect plants from pathogen [147]. This added benefit of disease and pest resistance further contributes to the overall effectiveness of bionanofertilizers in promoting healthy and resilient crops.

### Disadvantages of bionanofertilizers

Bionanofertilizers, which involve the use of nanotechnology in combination with biological components for enhancing plant growth and nutrient uptake, offer several potential advantages. However, like any emerging technology, they also come with certain disadvantages and concerns. Some of the disadvantages of bionanofertilizers include:

- The adoption and full use of the bionanofertilizer strategy in the agriculture sector cannot be achieved just through laboratory-based experiments. Therefore, to provide an accurate representation of the environmental impact of nanoparticles, an experimental design needs to be placed in a natural setting.
- Validating the acceptable and safety limit of nanoparticle doses should be done by scientific and government-based risk assessments. Moreover, handling the drawbacks of the organic waste used needs to be investigated and explained using realistic natural field settings.
- Gaining a thorough grasp of the toxicity of bionanofertilizer applications on plants requires an awareness of their biodegradability and biomagnification transfer effects.
- The development and use of bionanofertilizers may raise issues related to intellectual property rights and control over agricultural technologies. This can have implications for the concentration of power in the agricultural sector.

## Conclusions

Nanoparticles act as carriers for beneficial microbes and nutrients, delivering them directly to plant roots for improved uptake and efficiency. Bionanofertilizers can improve a plant’s tolerance to environmental stress factors like drought, salinity, and extreme temperatures. The nanocarriers can provide a slow and sustained release of nutrients and microbes over time, reducing the need for frequent applications. With targeted delivery and enhanced stress tolerance, bionanofertilizers have the potential to significantly improve crop yields. By minimizing reliance on chemical fertilizers and promoting sustainable practices, bionanofertilizers can contribute to a greener agricultural future.

Bionanofertilizers represent a significant step forward in biofertilizer technology. Researchers are working on creating bionanofertilizers that combine multiple beneficial properties, such as nitrogen fixation, nutrient solubilization, and biocontrol, into a single product. Bionanofertilizers can be integrated with precision agriculture techniques for customized application based on specific soil and crop needs. Advancements in nanotechnology can lead to bionanofertilizers with longer shelf life and improved efficiency. As research validates the benefits of bionanofertilizers and production costs decrease, their adoption by farmers is expected to rise significantly.

## Data Availability

No datasets were generated or analysed during the current study.
